# The Sonography and Physical Findings on Shoulder after Selective Neck Dissection in Patients with Head and Neck Cancer: A Pilot Study

**DOI:** 10.1155/2019/2528492

**Published:** 2019-07-22

**Authors:** Yu-Chi Huang, Yan-Yuh Lee, Hui-Hsin Tso, Po-Cheng Chen, Yi-Cun Chen, Chih-Yen Chien, Ya-Ju Chung, Chau-Peng Leong

**Affiliations:** ^1^Department of Physical Medicine and Rehabilitation, Kaohsiung Chang Gung Memorial Hospital and Chang Gung University College of Medicine, Kaohsiung, Taiwan; ^2^Department of Physical Medicine and Rehabilitation, Kaohsiung Chang Gung Memorial Hospital, Kaohsiung, Taiwan; ^3^Department of Otolaryngology, Kaohsiung Chang Gung Memorial Hospital and Chang Gung University College of Medicine, Kaohsiung, Taiwan

## Abstract

The aim of this study is to investigate soft tissue disorders of affected shoulders after nerve-sparing selective neck dissection (SND) in patients with head and neck cancers (HNCs) by sonography. Eighteen HNCs patients with shoulder disability after SND were enrolled. Shoulder motions, pain, and the sonographic findings were measured and analyzed. Significantly decreased flexion, abduction, and increased pain were found on affected shoulders compared with normal shoulders. There were significant decrease in thickness of trapezius muscle (p=0.001), abnormal findings of supraspinatus tendon (p=0.022), and subdeltoid bursa (p=0.018) on surgical side. The ratio of trapezius muscle atrophy was related to shoulder pain (p=0.010). Patients with subdeltoid abnormalities had significant limitation on shoulder flexion and abduction. Abnormalities of supraspinatus tendon and subdeltoid bursa on sonography and trapezius muscle atrophy may play a key role in shoulder pain and shoulder flexion and abduction limitations.

## 1. Introduction

Spinal accessory nerve (SAN) injury is a common comorbidity after neck dissection in patients with head and neck cancers (HNCs) [1, 2]. In the past, radical neck dissection [3] has been the standard surgical method for patients with HNCs with neck lymph node metastasis [4]. However, this technique led to complete SAN injury, which is associated with considerable ipsilateral shoulder pain and dysfunction, and might have negative impacts on life quality [5–8]. Therefore, to preserve the SAN, a more selective operation, the nerve-sparing technique of selective neck dissection (SND) was in turn developed to presumably limit the morbidity of the procedure based on the potential topographical tumor subsites-lymph node relationship, and it has become more popularly performed in patients with HNCs with no lymph node metastasis (N0) or limited metastasis (N1) [6, 9–11]. But, still, up to 67% of patients were reported to have injury to the accessory nerve after SND [2, 12].

The accessory nerve is the key nerve that innervates the sternocleidomastoid and trapezius muscles [13]. The major functions of the trapezius muscle are to maintain the scapula position and to aid shoulder abduction and flexion. Therefore, injury to the accessory nerve could cause weakness of the trapezius muscle, which is related to scapula malposition in depression, abduction, and medial rotation, as well as to reduced shoulder abduction and flexion [1, 14, 15]. After SND, shoulder droop was reported to occur in 13% of patients [2], and decreased active abduction range was seen in 5–25% of patients [16]. These biomechanical deficits lead to shoulder pain [11, 17, 18] and shoulder dysfunction [19], which are associated with reduced quality of life [7, 20] in long-term HNC survivors.

Clinically, functional active motion measurements of the shoulder, pain scales, shoulder functional tests, and quality of life questionnaires are usually used to assess the negative impacts on shoulder dysfunction of accessory nerve injury after surgery. Shoulder pain, shoulder dysfunction, and quality of life after surgery are influenced by the type of neck dissection performed [21]. Shoulder pain was reported to occur in 70% of patients with HNCs after neck dissection and before discharge [5]. Further, the incidence of shoulder pain at 6 months after surgery was still 29–31% and 36–56% in patients who underwent SND and modified neck dissection, respectively [12]. A review of the literature indicates that the pathogenesis of shoulder pain in patients with HNCs after SND has not been fully investigated. One study considered that adhesive capsulitis at the shoulder joint is a main contributing factor to shoulder pain after neck dissection in patients with HNCs [22]. In recent decades, musculoskeletal sonography (MS) has been a popular tool for detecting structural or soft tissue disorders related to shoulder pain. MS is a convenient tool for evaluating the surrounding muscle and tendon injury at the shoulder joint without radiation exposure. We considered that SND complicated with trapezius muscle atrophy may have some associations with soft tissue injuries of the shoulder leading to shoulder pain and dysfunction in patients with HNCs.

No study has investigated the soft tissue structures or rotator cuff condition of the affected shoulders after SND. Therefore, in this study, we aimed to use MS to investigate the injuries of surrounding soft tissues in affected shoulders and to determine the relationship between shoulder soft tissue injury and shoulder function after SND, at the subacute stage, in patients with HNCs.

## 2. Materials and Methods

### 2.1. Research Design

This study was a prospective cross-sectional study. A total of 18 patients with HNCs who developed shoulder pain and dysfunction after SND between April 2015 and December 2017 were enrolled. According to the medical history of each patient, the diagnosis, localization, and stage of HNCs were determined. For this study, we collected patients with HNCs who had undergone SND and were in stable condition, with well-healed skin, and without localized or systemic infection, or metastasis to the neck and shoulder. This study was approved by the institutional review board (approval no. 103-5312B). Written informed consent was obtained from all participants after the enrollment.

### 2.2. Participants

Patients with a history of HNCs were enrolled in this study. On the basis of the patients' clinical history, physical findings, imaging findings on computed tomography (CT) or magnetic resonance imaging (MRI) of the head and neck, and pathological records, the clinical diagnosis and stage of HNCs were determined. Because of motor dysfunction and pain around the shoulder, the patients were referred to the rehabilitation department from the HNC center. The inclusion criteria of this study were as follows: (1) age from 20 to 65 years, (2) a diagnosis of HNC and development of shoulder pain or movement dysfunction after nerve-sparing SND, and (3) <6 months duration since nerve-sparing SND. The exclusion criteria were as follows: (1) history of neuromuscular disease or tendinopathy on the affected shoulder that caused shoulder motion limitation and pain, (2) any active inflammation, skin infection, or soft tissue swelling at the affected neck, and (3) severe cognitive impairment before participation in the study.

A total of 18 patients were finally included in the study, and, of them, 14 patients underwent unilateral neck dissection and 4 patients underwent bilateral neck dissection. According to the surgical records, of the 4 patients who underwent bilateral operation, 2 patients not only additionally received neck dissection at the affected (major) side but also underwent removal of a few lymph nodes on the contralateral (minor) side. Therefore, we categorized the major operation side into the surgical group and did not take into account the minor side. In the other 2 patients, bilateral SNDs were performed; thus, we categorized both shoulders into the surgical group. Finally, the shoulders were allocated into 2 groups: 14 shoulders in the nonsurgical group and 20 shoulders in the surgical group. All the participants underwent level 2b dissection.

### 2.3. Measurements

The demographic information and clinical data of each participant were obtained from self-reports and medical history, which included sex, age, height, weight, surgical side, duration since the operation, DASH (disabilities of the arm, shoulder, and hand) questionnaire, shoulder pain, and tumor location. To quantify the severity of pain, the visual analogue scale (VAS) was used, in which 0 represents no pain and 10 means the worst pain. The objective physical measurements, including range of motion (ROM) of all plane motions of both shoulders (flexion, extension, abduction, adduction, and internal and external rotation), were assessed by one occupational therapist with a standard goniometer.

An experienced physician performed B-mode sonography (two-dimensional imaging scan) and sonoelastography of both shoulders for each patient during imaging assessment. All sonographic images were evaluated by the same physiatrist certified by the Taiwan Society of Ultrasound in Medicine. The physician used an MS device with a 9-14 MHz linear-array transducer (ACUSON S2000; Siemens, Malvern, PA, USA). The biceps tendon (long head), subscapularis tendon, supraspinatus and infraspinatus tendons, teres minor tendon, subdeltoid bursa, trapezius muscle, and deltoid muscle were investigated. On shoulder sonography, the echogenicity and hyperemia of each tendon, the fluids surrounding the tendons or in the bursa, and the thickness of the assessed muscles were evaluated by B-mode sonography and the elasticity of those muscles was measured using sonoelastography in this study. We measured the thickness of bilateral trapezius and deltoid muscles at the muscle belly in each patient. During sonoelastography, the participants sat upright and placed their arms on their thighs in a comfortable position. We repeatedly performed acoustic radiation force impulse and shear wave velocity (SWV) assessment 7 times at the muscle belly of each muscle and recorded the median value for further analysis.

### 2.4. Statistical Analysis

Statistical analysis was performed using SPSS 20.0 software (Statistics Standard 20.0; SPSS, Chicago, IL, USA). The number of patients by sex, surgical side, and cancer site was counted using a contribution table. Age, height, weight, duration since the operation, DASH score, and VAS scores were recorded and presented as medians and quartile 1 and quartile 3 values.

For between-group comparisons, we used the nonparametric Mann-Whitney U-test to analyze shoulder joint motions and muscle thickness on sonoelastography. Fisher's exact test was used for between-group comparisons of the findings of the tendons on shoulder sonography. For the ratio of trapezius atrophy, the thickness of the surgical side was divided by that of the nonsurgical side, and Spearman correlation was further used to analyze the relation between the ratio of muscle atrophy and the level of pain. On the basis of the diagnosis according to sonography results, we categorized the participants into 2 groups according to the presence or absence of abnormal findings on supraspinatus tendon or subdeltoid bursa. Then, the Mann-Whitney U-test was used to perform between-group comparisons of shoulder ROMs. A significant statistical difference was defined as p < 0.05.

## 3. Results

Table 1 presents the clinical characteristics of the patients. A total of 18 patients with HNC were enrolled and assessed: 17 of 18 patients were men (94.4%) and 1 patient was a woman (5.6%). The median age, height, and weight of these patients were 53 years, 167 cm, and 64.5 kg, respectively. Of the 18 patients, 14 (77.8%) underwent unilateral neck dissection (right/left 7:5) and 4 (22.2%) underwent bilateral neck dissection. The median duration since the operation was 2.6 months. More specifically, for participants who underwent radiotherapy, the median duration since the operation was 3.2 months, and yet for those without receiving radiotherapy, it was 2.4 months. The median DASH score was 12.5 in these patients with HNCs. The median VAS score for the affected shoulders was 4. In 17 of 18 patients (94.4%), the primary cancer developed in the oral cavity including the buccal area, tongue, gums, soft palate, and lip; however, in 1 patient, the cancer was located in the pharynx (5.6%). All participants in this study were right-hand dominant.

**Figure 1:**
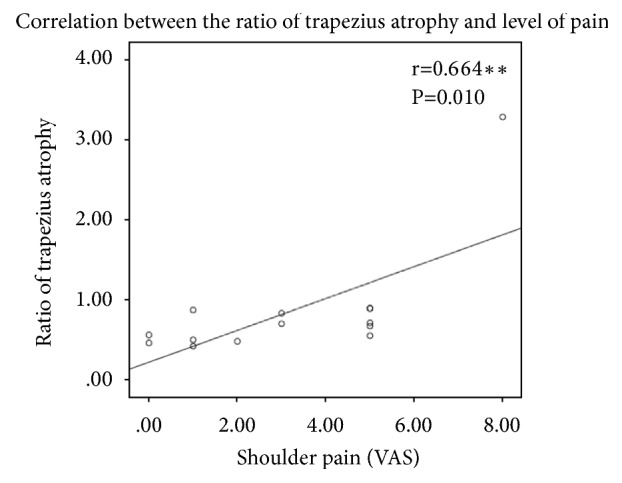
The correlation between the ratio of trapezius atrophy and the level of pain. Spearman correlation was used to analyze the relation between level of trapezius atrophy and pain. The X axis was the level of shoulder pain, which was assessed with VAS; the Y axis represented the ratio of trapezius atrophy that was calculated with the sonography results.

**Figure 2:**
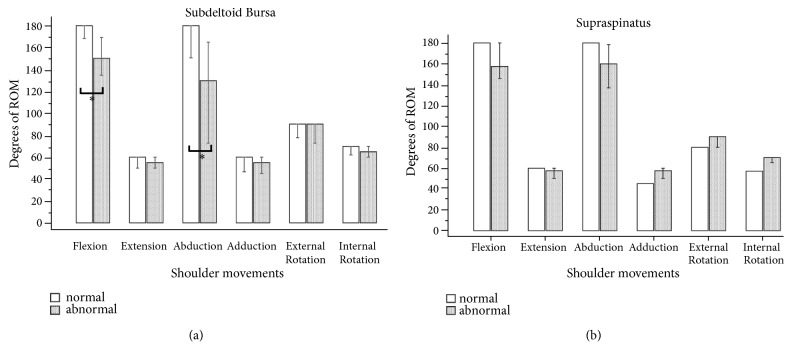
The between-group comparisons of shoulder movements based on sonographic findings of subdeltoid bursa (a) or supraspinatus tendon (b).

The results of shoulder pain and motion assessments are shown in Table 2. The median VAS score of shoulder pain in the surgical side was 4, which was significantly higher than that in the nonsurgical side (p = 0.001). The active ROMs (AROMs) of the normal and affected shoulders were evaluated and are shown in Table 2. The median AROMs of shoulder flexion in the surgical and nonsurgical sides were 147.5° and 180°, respectively. In shoulder abduction, the median AROM was 140° in the surgical side and 180° in the nonsurgical side. There were significant differences in shoulder flexion (p = 0.001) and abduction (p = 0.001) between the nonsurgical and surgical sides. The median muscle thickness of the trapezius muscle was 0.72 cm in the surgical side and 1.18 cm in the nonsurgical side, whereas that of the deltoid muscle was 1.82 cm in the surgical side and 1.8 cm in the nonsurgical side (Table 3). There was a significant difference in trapezius muscle thickness between the surgical and nonsurgical sides (p = 0.001). The association between the ratio of trapezius atrophy and the level of shoulder pain was moderate (R = 0.664, p = 0.010) (Figure 1). Significant differences in the existence of abnormal findings were also noted in the supraspinatus tendon (p = 0.022) and subdeltoid bursa (p = 0.018) between the affected and normal shoulders. Moreover, we found that patients with abnormal findings in the subdeltoid bursa had significant limitation in shoulder flexion and abduction (Figure 2).

## 4. Discussion

To our knowledge, this is the first study to investigate soft tissue injuries of the shoulders in patients with HNCs who developed shoulder pain and dysfunction after SND, by using MS and sonoelastography. Our findings also revealed that there were significant shoulder pain and shoulder motion limitations while performing flexion and abduction after SND in patients with HNCs. In addition, significant muscle atrophy at the trapezius muscle and higher prevalence of supraspinatus tendon injury and subdeltoid bursitis or effusion on the affected shoulders were found using MS. We also found significantly decreased motions of shoulder flexion and abduction in patients with HNCs with subdeltoid bursitis or effusion on the affected shoulder.

The surgical method or the type of neck dissection was a major contributing factor to shoulder pain, shoulder motor dysfunction, and quality of life [21]. SND has been reported to cause less shoulder dysfunction; however, a certain proportion of patients still experienced shoulder impairment, including pain and ROM limitation [21, 23]. Erisen et al. reported that the SAN function was electrophysiologically impaired after neck dissections surgery even if the nerve was preserved [24]. Weakness of the trapezius muscle may be the first sign after a minor trauma in the SAN, which interferes with the abduction and flexion of the shoulder joint, and leads to scapula malposition and displacement [25]. Therefore, to maintain the stability of the scapula and support shoulder motion, more shoulder muscles need to be involved to compensate for the motion dysfunction, which may cause further soft tissue injury in the affected shoulder after the surgery. Moreover, some authors reported that shoulder pain after SND was related to nerve palsy or injury at the acute stage but was predominantly associated with adhesive capsulitis of the affected shoulders in the chronic stage [22]. In this study, we used MS to assess and investigate the surrounding soft tissue conditions of the painful shoulders in patients with HNCs after SND, at the subacute stage of injury. We found not only trapezius atrophy and moderate pain in the affected shoulders, but also a higher prevalence of supraspinatus tendon and subdeltoid bursa inflammation on sonography in patients who underwent SND surgery 2–3 months ago. Furthermore, this study found that patients with HNCs with subdeltoid bursitis or effusion after SND had significant motion limitations in shoulder flexion and abduction. The subdeltoid bursa is located between the deltoid muscle and the supraspinatus tendon, and it helps reduce friction in the shoulder joint. Moreover, trapezius weakness leading to scapular instability and impaired scapulohumeral rhythm causes shoulder impingement syndrome, which is related to bursitis or rotator cuff tendinosis [26, 27]. Shortly summed up, the trapezius muscle weakness following neck dissection would cause biomechanical changes, which leads to shoulder impingement and is associated with subdeltoid bursitis and supraspinatus tendinopathy. These pathogeneses may be potential contributing factors to further shoulder dysfunction and pain. Therefore, we believe that early exercise intervention for improving trapezius muscle strength could prevent subsequent shoulder soft tissue injury and reduce shoulder dysfunction and pain in patients after SND surgery.

SAN injury after neck dissection is strongly associated with subsequent trapezius muscle atrophy. On shoulder sonography and sonoelastography, we found significantly decreased thickness at the muscle belly of the trapezius with lower SWV in the surgical side in patients with HNCs after SND surgery. The sonographic finding of the trapezius muscle is compatible with trapezius muscle atrophy and weakness reported in previous studies. In addition, we applied sonoelastography to detect the stiffness condition of the atrophied trapezius muscle, and we found significantly lower SWV values due to the intrinsic and physiological changes of the muscle properties related to muscle atrophy and fatty infiltration of the muscle. We found that sonoelastography is a useful imaging tool for the objective evaluation of the mechanical properties of atrophied skeletal muscle.

This study has several limitations. As it was a cross-sectional study, there were no long-term follow-up data. Prospective longitudinal studies may provide better insight on the effects of the operation on patients. There was no control group to compare the differences between affected shoulders with or without having neck dissection. This study focused only on the functional assessment of the shoulder and upper limb; it lacked an objective assessment of the soft tissue conditions around the shoulder girdle. Imaging studies may provide better information related to musculoskeletal disability. Besides, some other important aspects such as quality of life were not included in this study. In the future, those limitations should be considered.

## 5. Conclusion

In summary, moderate shoulder pain and shoulder motion limitations occur in patients with HNCs after SND. Musculoskeletal sonography is a convenient tool to objectively detect trapezius atrophy and rotator cuff disorders in those patients. Surrounding soft tissue injuries, especially in the supraspinatus tendon and subdeltoid bursa, of the affected shoulders with trapezius muscle atrophy may be a key contributor to shoulder pain and shoulder flexion and abduction limitations. We believe that shoulder exercises and postural education may prevent further shoulder disorders in patients with HNCs after SND.

## Figures and Tables

**Table 1:** Clinical characteristics of enrolled participants.

	Average
Gender, male/female, (n)	17; 1
Age, years (median (IQR))	53(47.5-57)
Height, cm (median (IQR))	167(162.75-172)
Weight, kg (median (IQR))	64.5(55-75.1)
Operated side, right/left, (n)	11; 9
Cancer site, (n)	
Oral cavity (buccal areas, tongue, gums, soft palate, and lip,)	17
Pharynx	1
Duration after operation, months (median (IQR))	2.6(1.87-3.35)
RT(+), (n), months (median (IQR))	11, 2.4(1.65-3.15)
RT(-), (n), months (median (IQR))	7, 3.2(2.35-4.05)
DASH, (median, IQR)	12.5(3.1-27.29)

Descriptive statistics was used for calculating the number of genders, surgical side, and tumor site. Frequency distribution table was used for calculating the median number of age, height, weight, and duration after operation.

DASH = The disabilities of the arm, shoulder and hand; VAS = Visual Analogue Scale.

**Table 2:** Comparison of pain level and range of motion of shoulder joint between surgical and nonsurgical sides.

	Surgical side (n=20)	Nonsurgical side (n=14)	P
VAS, (median, IQR)	4(1-5)	0(0-0)	0.001*∗*
Range of Motion, degree (median, IQR)			
Flexion	147.5(135-158.75)	180(180-180)	0.001*∗*
Extension	55 (50-60)	60(50-60)	0.112
Abduction	140 (71.25-160)	180(180-180)	0.001*∗*
Adduction	55 (45-60)	60(45-60)	0.569
Internal rotation	70 (60-70)	70(60-71.25)	0.931
External rotation	87.5(71.25-90)	90(77.5-90)	0.396

Mann-Whitney U test was used for the between-group comparison of range of motion of shoulder joint.

VAS = Visual Analogue Scale.

*∗*p<0.05.

**Table 3:** Comparison of sonographic findings between surgical and nonsurgical side.

	Surgical side (n=20)	Nonsurgical side (n=14)	P
Muscle thickness, cm (median (IQR))			
Trapezius	0.72(0.60-1.03)	1.18(1.08-1.31)	0.001*∗*
Deltoid	1.82(1.62-2.04)	1.8(1.54-2.09)	0.986
ARFI with SWV, m/s (median (IQR))			
Trapezius	3.05(2.35-3.30)	3.2(3.10-3.45)	0.033*∗*
Deltoid	2.63(2.41-2.93)	2.73(2.63-3.06)	0.090
Abnormal findings od sonography, (n, %)			
Biceps (tenosynovitis, tendinosis, tear)	8(40.0%)	2(14.3%)	0.107
Subscapularis(tendinosis, tear)	16(80.0%)	10(71.4%)	0.428
Supraspinatus(tendinosis, tear)	20(100.0%)	10(71.4%)	0.022*∗*
Infraspinatus(tendinosis, tear)	1(5.0%)	1(7.1%)	0.661
Teres Minor(tendinosis, tear)	1(5.0%)	0(0)	0.588
Subdeltoid Bursa(effusion, bursitis)	11(55.0%)	2(14.3%)	0.018*∗*

Frequency distribution table was used to calculate the total number of each diagnosis. Fisher exact test was used for the between-group comparison of each sonographic finding. Mann-Whitney U test was used for between-group comparison of muscle thickness of trapezius and deltoid muscle belly.

*∗*p<0.05.

ARFI= acoustic radiation force impulse; SWV= shear wave velocity.

## Data Availability

The data used to support the findings of this study are available from the corresponding author upon request.
